# Neuroprotective Effect of Melatonin: A Novel Therapy against Perinatal Hypoxia-Ischemia

**DOI:** 10.3390/ijms14059379

**Published:** 2013-04-29

**Authors:** Daniel Alonso-Alconada, Antonia Álvarez, Olatz Arteaga, Agustín Martínez-Ibargüen, Enrique Hilario

**Affiliations:** Department of Cell Biology and Histology, School of Medicine and Dentistry, University of the Basque Country, Barrio Sarriena s/n, Leioa 48940, Bizkaia, Spain; E-Mails: antoniaangeles.alvarez@ehu.es (A.A.); olatz_b@hotmail.com (O.A.); agustin.martinez@ehu.es (A.M.-I.); enrique.hilario@ehu.es (E.H.)

**Keywords:** melatonin, neuroprotection, hypoxia-ischemia, newborn

## Abstract

One of the most common causes of mortality and morbidity in children is perinatal hypoxia-ischemia (HI). In spite of the advances in neonatology, its incidence is not diminishing, generating a pediatric population that will require an extended amount of chronic care throughout their lifetime. For this reason, new and more effective neuroprotective strategies are urgently required, in order to minimize as much as possible the neurological consequences of this encephalopathy. In this sense, interest has grown in the neuroprotective possibilities of melatonin, as this hormone may help to maintain cell survival through the modulation of a wide range of physiological functions. Although some of the mechanisms by which melatonin is neuroprotective after neonatal asphyxia remain a subject of investigation, this review tries to summarize some of the most recent advances related with its use as a therapeutic drug against perinatal hypoxic-ischemic brain injury, supporting the high interest in this indoleamine as a future feasible strategy for cerebral asphyctic events.

## 1. Introduction

The main function of the pineal gland in all species is to transduce information concerning light-dark cycles to body physiology, particularly for organization of body rhythms via its main hormone melatonin [1]. Based on the ability of melatonin (*N*-acetyl-5-methoxytryptamine) and its metabolites to scavenge a wide variety of free radicals (FR), it is not surprising to consider it as one of its most important functions in living organisms leading to protect them from oxidative stress [2,3]. Acting as a direct scavenger, this neurohormone is able to remove FR, such as singlet oxygen, superoxide anion radical, hydroperoxide, hydroxyl radical and the lipid peroxide radical [2,4]. Moreover, a single melatonin molecule may generate products in a scavenger cascade, which may collectively eliminate up to ten FR [4]. Melatonin can develop indirect antioxidant actions through the improvement of the mitochondrial efficiency [5], the stimulation of the gene expression and the activation of some of the most important antioxidant enzymes, including superoxide dismutase (SOD), catalase, glucose-6-phosphate dehydrogenase, glutathione reductase and glutathione peroxidase [6] and also with the strengthening of the antioxidant effect of substances, like glutathione, vitamin E and vitamin C [7].

The brain is particularly sensitive to FR damage due to its high utilization of oxygen, its relatively poorly developed antioxidant defense and its high amount of easily oxidizable fatty acids. Thus, the use of melatonin as pharmacological agent against neurodegenerative disorders, such as Huntington’s disease, Alzheimer’s disease and Parkinsonism and also against ischemic brain injury/stroke, has been extensively evaluated. With an incidence of 2–6/1,000 term births [8], perinatal hypoxia-ischemia (HI) remains the single most important cause of brain injury in the newborn, leading to death or lifelong disability [9,10]. Despite the improvements in perinatal care, significant neurological sequelae can occur in as many as 50%–75% of these asphyctic children, which may suffer from long-term neurological consequences, such as cerebral palsy, mental retardation and epilepsy [11–13]. Asphyxia is also associated with attention deficits and hyperactivity in children and adolescents [14,15]. During the last decade, melatonin has started to be considered as an attractive option in order to minimize as much as possible the neurological sequelae from hypoxic-ischemic brain injury. It easily crosses both the placental and the blood-brain barrier, reaching subcellular compartments with a low toxicity and high efficacy [16–18], and even at high supra-physiological concentrations, there appear to be no adverse side-effects [19], making it a relatively-safe therapy that could be administered to babies.

## 2. Brain Protection

Following cerebral asphyxia, HI starts out a multi-faceted cascade of events that ultimately causes cell death and often damages the whole brain [20]. Everything begins when the reduction in oxygen and blood supply induces a decrease in oxidative phosphorylation and the neonate’s brain converts to anaerobic metabolism in an effort to sustain functional ability. Anaerobic metabolism leads to a rapid depletion of ATP, accumulation of lactic acid and failure of ion pumps, resulting in a massive entry of sodium, calcium and water into the cells. Afterwards, multiple and diverse downstream biochemical reactions aggravate the pathogenesis of hypoxic-ischemic brain damage, being the most important, among others, the production of reactive oxygen species leading to oxidative stress, the massive increase in free cytosolic calcium concentrations and the drop in mitochondrial function triggering the activation of apoptotic pathways, DNA fragmentation and cell death. After ischemic brain injury/stroke, melatonin has showed a remarkable capacity to reduce infarct volume and/or inhibit neuronal cell death after in different mammalian species and using different experimental models [21–33].

**Figure 1:**
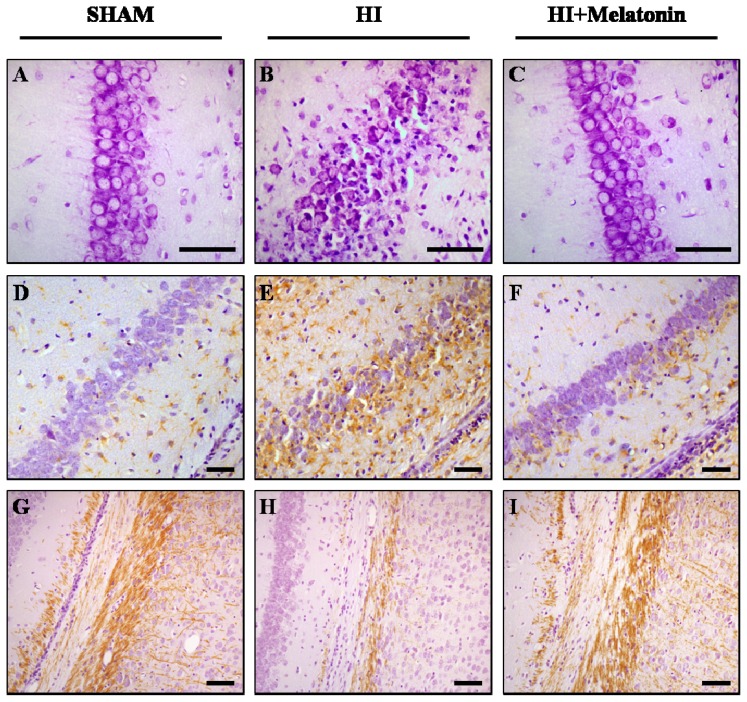
Nissl-stained (**A**–**C**), myelin basic protein (**D**–**F**) and glial fibrillary acidic protein (**G**–**I**) immunolabeled brain sections corresponding to the surrounding areas of the CA1 region of the hippocampus and the external capsule showing cell loss (**B**), myelination deficit (**E**) and reactive gliosis (**H**) after hypoxia-ischemia and recovery after melatonin administration. Seven-day old rats were subjected to hypoxia-ischemia (left common carotid artery ligated and then 8% oxygen for 2 h) and sacrificed seven days after the injury. Pups without ischemia or hypoxia served as controls (Sham group). Bar: 100 μm.

Melatonin administration after neonatal HI has been shown to reduce infarct volume both administered before or after the injury [34–36]. Indeed, melatonin was able to decrease sensorimotor asymmetry and learning deficits, thus protecting the pups from the long-term consequences of neonatal asphyxia [34]. While virtually every cell is affected by asphyxia, they do not respond in the same way during HI, being neurons the most sensitive cells to the lack of oxygen and showing a selective vulnerability [37–39]. Histological analysis demonstrated an increase in the number of morphologically well preserved neurons in melatonin-treated animals in the CA1, CA2–CA3 areas and dentate gyrus of the hippocampus and parietal cortex when compared with the hypoxic-ischemic group [33,40,41] (Figure 1). Even though astrocytes are more resistant than neurons to oxygen deprivation, their death may give rise to a new wage of neuronal death due to their role in the maintenance of the homeostatic state of neurons. Therefore, astrocytes can modulate in a significant manner the extension and degree of severity of the damage [42,43], either conferring neuroprotection by scavenging reactive oxygen species and also assisting with reconstruction from brain injury [44] or leading to deficiencies in the myelination processes, neuronal signaling impairment and an increase the inflammatory response [45,46]. Melatonin has demonstrated to reduce the expression of the glial fibrillary acidic protein [33] (Figure 1), whose accumulation is related with the creation of new astrocytic processes and reactive gliosis [43]. In addition to neurons, oligodendrocytes are particularly vulnerable to asphyxia, affecting myelination that gives rise to white matter lesions and damaging gray matter oligodendrocyte progenitors [47]. An abnormal decrease in the expression of myelin basic protein leading to myelination deficit is considered hallmark of inflammation-associated diffuse white matter damage [48,49]. In this sense, several groups have suggested that melatonin may be of therapeutic value in ameliorating hypoxic-ischemic damage to the developing white matter through normalization of the myelination process [33,50–52] (Figure 1).

## 3. Antioxidant

The high incidence of hypoxic-ischemic brain lesions in newborns can be partly attributed to the fact that the developing brain is especially vulnerable to oxidative stress. After neonatal asphyxia, reperfusion brings about the overproduction of FR leading to oxidative stress, as the antioxidant capacity of immature neurons is easily overwhelmed by hypoxia-induced reactive oxygen species. The newborn brain is especially vulnerable to oxidative imbalance, due to its increased fatty acid content, higher concentrations of free iron, high rates of oxygen consumption, low concentrations of antioxidant, an imbalance of antioxidant enzymes, as for example catalase CuZn-SOD-1, mitochondrial SOD-2 and glutathione peroxidase and oxygen-induced vasoconstriction, leading to reduced brain perfusion, among others [53–55].

When membrane lipoproteins and polyunsaturated fatty acids suffer attacks from FR, many oxygenated compounds, particularly aldehydes, such as malondialdehyde (MDA), are produced. Thus, the evaluation of lipid peroxidation is a useful tool to evaluate oxidative stress leading to brain damage. Melatonin has been able to abolish lipid peroxidation in late-gestation fetal sheep in respond to umbilical cord occlusion [56] and to avoid the rise in MDA induced by hypoxia in rat pups [57] and asphyxiated human newborns [58]. Deferoxamine-chelatable free iron, isoprostanes, neuroprostanes and neurofurans are also quantitative biomarkers of oxidative damage [59–61], and after melatonin administration, their levels were significantly lower than those in hypoxic-ischemic rats [62,63]. These results were similar to those observed in fetal sheep after umbilical cord occlusion, where the production of 8-isoprostanes was attenuated [64]. On the other hand, melatonin may prevent protein oxidation in the brain tissue of hypoxic neonatal rats [65], as terminal products of protein exposure to FR are considered reliable markers of the degree of protein damage in oxidative stress [66]. Additionally, the activity of the antioxidative enzyme catalase is maintained [57], hydroxyl formation reduced [56] and nitrite/nitrate levels reduced [58] in different animals models subjected to hypoxia.

## 4. Anti-Apoptotic

Under pathophysiological conditions, one of the most important key regulators of apoptotic cell death is mitochondrial impairment, as the disruption of its membrane integrity and loss of membrane potential can determinate cell survival by overproduction of reactive oxygen species, abnormal calcium homeostasis and release of apoptotic proteins. In several studies demonstrating anti-apoptotic actions, melatonin prevented cytochrome *c* release [32,67,68], reduced or blocked caspase-1 and caspase-3 activation [32,67,69–73], increased the expression of anti-apoptotic proteins Bcl-2 [71,74,75] and Bcl-xL[70], diminished Bad [31,72] and Bax [71] pro-apoptotic proteins, inhibited poly-ADP-ribose-polymerase cleavage [72], avoided mitochondrial permeability transition pore opening, thus counteracting the collapse of the mitochondrial membrane potential [67,69], and decreased the number of TUNEL-positive cells/DNA breaks [31,32,72,75–78]. In the central nervous system, melatonin can also generate anti-excitatory effects on neurons through the modulation of gamma-aminobutyric acid and glutamate receptors [79,80], inducing a decrease in cytosolic calcium concentrations [81,82].

Shortly after a hypoxic-ischemic event, reactive oxygen species overproduction can start out a harmful multi-faceted cascade that includes lipid peroxidation, protein oxidation and DNA fragmentation, ultimately damaging vital cellular components as nucleic acids, cell membranes and mitochondria, resulting in subsequent cell death in the immature brain [83–85]. In this regard, we have recently shown that HI can develop a widespread increase in reactive oxygen species after perinatal asphyxia, an overproduction correlated with the number of, as well as with the distribution of apoptotic cells [86].

Melatonin may be an effective prophylactic agent for use in late pregnancy to protect against mitochondrial-induced cell death after a hypoxic-ischemic event at birth. Given to pregnant rats, it prevented oxidative mitochondria damage after ischemia-reperfusion in premature fetal rat brain [87] by means of the maintenance of the number of intact mitochondria and the respiratory control index (an indicator of mitochondrial respiratory activity), as well as the reduction in thiobarbituric acid-reactive substances concentration (a marker of oxidative stress) [40,41]. Hutton *et al.* studied caspase-3 activation and fractin in order to evaluate the anti-apoptotic effect of melatonin in a model of birth asphyxia in the spiny mouse, showing lower levels after its administration [88]. Accordingly, Fu *et al.* demonstrated the inhibition of caspase-3 activation, the induction of Bcl-2 expression and the increase Bcl-2/Bax ratio in a model of hypoxia *in vitro* [89]. Melatonin administration not only generates a neuroprotective effect when administered before the hypoxic-ischemic event, but also when given after the onset of the injury. Using the terminal deoxynucleotidyl transferase dUTP nick end labeling method (TUNEL) to detect DNA fragmentation and apoptotic figures, melatonin-treated neonatal animals have shown a reduction in TUNEL-positive cells per unit area in neonatal sheep [64] and in rats [33,35,36].

As shown above, melatonin can exert a wide range of antiapoptotic effects, mainly targeting mitochondria, but it can also enhance cell survival pathways leading to cell rescue. For instance, protection from cerebral ischemic injury was attributed to the maintenance of signaling via the MAP kinase pathway, leading to the prevention Bad dephosphorylation [31]. Furthermore, melatonin can target PI3K/Akt pathway [70,72,90–92], mTOR [93] or the forkhead transcription factor, pAFX [91], and also restore JNK1/2 and ERK 1/2 phosphorylated levels [72,90], thereby preventing the proapoptotic actions of the dephosphorylated proteins.

## 5. Anti-Inflammatory

In recent years, the use of melatonin has started to be considered as another meaningful tool against inflammatory response in an effort to improve the clinical course of illnesses, which have an inflammatory etiology. The strategy mediated by melatonin and its main metabolites 6-hydroxy, *N*1-acetyl-*N*2-formyl-5-metho, *N*1-acetyl-5-methoxykynuramine and cyclic 3-hydroxy melatonin, encompasses the downregulation of some inflammation-related molecules, such as cytokines interleukin-6, interleukin-8 and tumor necrosis factor-α [94–100], 5-lipoxygenase [101], cyclooxygenase [102,103] and prostaglandin [104–106], an important reduction of nitric oxide (NO) and MDA levels [107] and also the inhibition of neuronal (nNOS) [108–111] and inducible (iNOS) nitric oxide synthases [111,112]. Even though NO participates in diverse processes acting as a physiological messenger, an excess in its concentrations can induce energy depletion, liberation of excitotoxic amino acids and a high ability to react with other FR. Downregulation of nNOS may contribute to the maintenance of electron transport chain function [109,110,113–115], thus protecting from NO-mediated mitochondrial impairment and cell damage [116–119]. From its part, counteraction to iNOS can avoid lipid peroxidation, shifts the glutathione redox state and boosts energy efficiency and ATP production in mitochondria [118,120,121]. Regarding the field of ischemic brain injury, melatonin and its metabolites have been able to reverse the inflammatory response and edema after stroke suppressing the production of inflammatory cytokines [102,122,123], reducing NOS [124], preventing the translocation of NF-κB to the nucleus [125,126] and decreasing cyclooxygenase-2 gene expression [127], molecular changes correlated with a reduction in the size of brain infarcts.

After perinatal asphyxia, FR also stimulate ischemic cells to secrete inflammatory cytokines and chemokines, which in turn can generate a wide variety of cytotoxic agents, including more cytokines, matrix metalloproteases, NO and more reactive oxygen species. These molecules can dismantle both blood brain barrier and extracellular matrix, allowing the blood, soluble elements and peripheral inflammatory cells to penetrate the brain, resulting in the exacerbation of the damage. Melatonin may be beneficial, as it reduces NO production, vascular endothelial growth factor concentration and, hence, vascular permeability that results increased after hypoxic exposure [128]. Prophylactic maternal treatment with melatonin has also demonstrated a reduction in central nervous system inflammation, by limiting macrophage infiltration and glial cell activation in a model of birth asphyxia in the spiny mouse [88]. Indeed, a reduced number of ED1 positive cells, a marker of activated microglia-macrophages, was found in neonatal rats treated with melatonin when comparing with pups without treatment [63].

## 6. Conclusions

Nowadays, there are convincing evidences demonstrating that melatonin treatment is highly effective against hypoxic-ischemic brain injury in different animal models by reducing infarct volume and neuronal loss, minimizing lipid and protein peroxidation, blocking some apoptotic pathways, inhibiting FR production and decreasing inflammation. Melatonin supplementation, which has a benign safety profile, may help to reduce complications in the neonatal period that are associated with short gestation [129] and has demonstrated not only neuroprotective actions against HI in animal models, but also in preliminary clinical trials [57,130–132].

Nevertheless, the complexity of neonatal hypoxic-ischemic pathophysiology determines that successful neuroprotection could be achieved only by multi-therapeutic approaches and optimizing therapy for neonatal brain injury will require capitalizing on multiple pathways, which prevent cell death. The use of synergic strategies, such as the association between hypothermia and other therapeutic drugs, may lead to a larger neuroprotective effect on the brain thus improving the neonatal outcome. In this regard, Robertson *et al.* have recently shown that melatonin administration to newborn piglets augments hypothermic neuroprotection by improving cerebral energy metabolism and by reducing brain damage [133].

Melatonin’s protective actions include not only its direct free radical scavenging, but also the interaction of its receptors and several yet-undefined functions, so the mechanisms underlying its neuroprotective benefits are not yet fully elucidated. Moreover, its variable oral absorption and rapid metabolization [134–136], the search for an appropriate dosage to obtain an antioxidant effect without desensitize melatonin receptors and its different pharmacokinetic profile when comparing preterm infants with adults (the half-life of melatonin in neonates is approximately 15 h, while in adults, it is around 45–60 min) [137], highlight the work that needs to be done before melatonin comes into clinical practice in a neonatal or pediatric critical care unit.

## Figures and Tables

**Table:** Summary of the experimental evidence regarding the beneficial effects of melatonin.

Target	Effect	References
Brain Protection		
Infarct volume	↓	[34–36]
Sensorimotor asymmetry	↓	[34]
Learning deficits	↓	[34]
Morphologically well preserved neurons	↑	[33,40,41]
GFAP expression	↓	[33]
MBP expression	↑	[33,50–52]
Antioxidant		
Lipid peroxidation and MDA production	↓	[56–58]
Iso- and neuroprostanes and neurofurans	↓	[62–64]
Protein oxidation	↓	[65]
Catalase’s activity	→	[57]
Hydroxyl formation	↓	[56]
Nitrite/nitrate levels	↓	[58]
Anti-apoptotic		
Cytochrome c release	↓	[32,67,68]
Caspase-1 and Caspase-3 activation	↓	[32,67,69–73,88,89]
Bcl-xL and Bcl-2 expression	↑	[70,71,74,75,89]
Bax expression	↓	[71]
Poly-ADP-ribose-polymerase cleavage	↓	[72]
Mitochondrial transition pore opening	↓	[67,69]
TUNEL-positive cells/DNA breaks	↓	[31–33,35,36,64,72,75–78]
Cytosolic calcium concentrations	↓	[81,82]
Oxidative mitochondria damage	↓	[87]
Mitochondrial respiratory activity	→	[40,41]
Oxidative stress	↓	[40,41]
Fractin levels	↓	[88]
Bcl-2/Bax ratio	↑	[89]
MAP kinase, JNK1/2 and ERK 1/2	→	[31,72,90]
Bad dephosphorylation	↓	[31]
Anti-inflammatory		
Interleukin-6, Interleukin-8 and Tumor Necrosis Factor- α	↓	[94–100,102,122,123,125,126]
5-lipoxygenase and Cyclooxyenase-2	↓	[101–103,127]
Prostaglandin	↓	[104,106]
NO, nNOS, iNOS and VEGF	↓	[107–112,124,128]
Macrophage infiltration	↓	[88]
ED1 positive cells	↓	[63]
